# Effects on Physicochemical, Nutritional, and Quality Attributes of Fortified Vegan Muffins Incorporated with Hempseed as an Alternative Protein Source

**DOI:** 10.3390/foods14040601

**Published:** 2025-02-11

**Authors:** Huimin Du, Inha Baek, Yunju Jang, Nurul Saadah Said, Won Young Lee

**Affiliations:** 1 School of Food Science and Technology, Kyungpook National University, Daegu 41566, Republic of Korea; duhuimin1998@naver.com (H.D.); ggotari@naver.com (I.B.); yunju@knu.ac.kr (Y.J.); nurulsaadah.said@gmail.com (N.S.S.); 2 Research Institute of Tailored Food Technology, Kyungpook National University, Daegu 41566, Republic of Korea

**Keywords:** hempseed protein, protein fortification, vegan muffin, baking properties, antioxidant activity, sensory evaluation

## Abstract

This study investigated the potential of hempseed protein isolate (HPI) as a protein fortifier and wheat flour substitute in vegan muffins. HPI was incorporated at 0% (CON), 10% (HP10), 20% (HP20), and 30% (HP30) substitution levels, and muffins were evaluated for physicochemical, functional, and sensory properties. Protein content significantly increased from 9.61% (CON) to 19.40% (HP30), while baking loss decreased from 21.33% to 19.77%, reflecting HPI’s superior water-holding capacity. Texture analysis showed hardness decreased from 179.72 g/cm^2^ (CON) to 137.73 g/cm^2^ (HP30), resulting in softer muffins with higher chewiness. This correlated with a more aerated crumb structure and smoother surface at higher HPI levels, indicating improved structural integrity. Rheological analysis revealed increased batter viscosity and shear-thinning behavior with HPI fortification. FTIR analysis exhibited redshifts in the Amide I and Amide II bands, suggesting enhanced protein–protein interactions and hydrogen bonding in fortified HPI muffins. Antioxidant activity increased significantly, with ABTS radical scavenging values rising from 32.66% (CON) to 46.28% (HP30), attributed to the bioactive peptides and phenolic compounds (144.67 mg GAE/g) in HPI. However, in vitro protein digestibility (IVPD) decreased from 66.08% to 42.63% due to protein–starch–lipid interactions inhibiting hydrolysis. Sensory evaluation showed no significant differences in aroma, taste, mouthfeel, or overall acceptability, with scores between 4.83 and 5.33 among all samples. These results demonstrate that HPI incorporation of up to 30% significantly enhances the nutritional profile, antioxidant activity, and textural properties of vegan muffins while maintaining overall sensory quality, supporting HPI’s potential as a sustainable protein fortifier in plant-based bakery products.

## 1. Introduction

Hemp (*Cannabis sativa* L.) is an herbaceous annual plant of the Cannabaceae family, native to the Eurasian region. Cultivated for over six thousand years for food, fiber, and medicinal purposes [1], hemp has a nutrient-dense seed profile containing approximately 35.5% oil (rich in polyunsaturated fatty acids), 25% protein, 20–30% carbohydrates, 28% total fiber (5.4% digestible and 22.2% non-digestible), and 5.6% ash [2]. In addition to their rich nutrient profile, hemp seeds are a source of phytocannabinoids, particularly cannabidiol (CBD), which exhibits notable biological effects. Of the many phytocannabinoids identified in Cannabis, CBD and tetrahydrocannabinol (THC) are the most well-known [3]. Unlike THC, which is psychoactive and primarily found in marijuana, CBD is abundant in industrial hemp and is non-psychoactive. Research has highlighted CBD’s therapeutic potential in treating various conditions, including inflammation, anxiety, arthritis, and neurodegenerative diseases [3,4,5].

Hempseeds are considered one of the most nutritionally complete plant-based foods and are widely accepted for human consumption. They can be consumed in various forms, including whole, hulled, or dehulled seeds, and are commonly processed into products such as hemp oil, nuts, and cakes. Processing methods like germination and sprouting have been shown to improve the phytochemical profile of hemp seeds, increasing concentrations of anti-inflammatory compounds (e.g., cannflavins A and B), polyphenols, flavonoids, and overall antioxidant activity, while also enhancing protein content under specific conditions [6,7]. Hempseeds are primarily processed to extract edible oil, leaving behind hemp cake as a by-product. A major by-product of hempseed oil extraction is hemp cake, which is often overlooked but remains a rich source of protein, fiber, and bioactive compounds. In alignment with sustainable food production practices, the upcycling of hemp cake into high-value ingredients offers a promising solution for reducing food waste while enhancing product nutrition. Hemp protein isolate (HPI), extracted from hemp cake, presents an innovative opportunity to fortify food products with plant-based protein. This strategy not only boosts the nutritional profile of foods but also contributes to economic and environmental sustainability by minimizing waste and supporting more circular food systems [8].

Hempseed protein, derived from hemp meal, offers a promising solution to the nutritional and functional challenges in plant-based food formulations. Hemp seeds are a nutrient-dense source of protein, containing high-quality protein that includes all nine essential amino acids [9]. The primary proteins in hempseed, edestin and albumin, are highly digestible and absorbable, distinguishing hempseed protein as a valuable alternative to other plant-based proteins [10]. In particular, its arginine content is significantly higher than other plant-based proteins [11], and it also contains higher levels of sulfur-containing amino acids than soy protein, the most commonly used plant-based protein. This makes hempseed protein a viable alternative for individuals with soy allergies and enhances its potential as a protein supplement in vegan diets [12]. Beyond its nutritional attributes, hemp protein exhibits excellent functional properties such as water- and oil-holding capacities, as well as foaming and emulsifying abilities, which make it highly versatile for various food applications, including baked goods, snack bars, and protein supplements [13]. Hemp protein also provides notable health benefits. Its high arginine content is associated with cardiovascular health, while its significant fiber content (27–36 g/100 g in whole seeds) supports digestive health and may lower the risk of colorectal cancer and cardiovascular disease [13,14]. Additionally, hemp protein is also rich in polyphenols and flavonoids, contributing to its antioxidant properties, which enhance its potential as a functional food ingredient [15].

In recent years, interest in vegan diets has grown rapidly due to the increasing emphasis on making health-conscious and environmentally sustainable food choices. This trend has led to significant growth in the vegan food market, presenting the challenge of developing nutritionally balanced products [16]. According to Parker and Vadiveloo [17], vegetarians often have lower protein intake compared to non-vegetarians. Inadequate protein intake in vegan diets can result in issues such as stunted growth in children and reduced muscle mass in adults [18]. Therefore, protein-fortified products play a crucial role in addressing these nutritional deficiencies. Vegan diets exclude all animal products, relying on plant-based protein sources such as grains, nuts, oilseeds, and legumes. However, plant proteins generally suffer from poor digestibility and low absorption rates due to their unbalanced amino acid composition and complex molecular structures [19,20].

Vegan products, particularly baked goods, face challenges not only in achieving adequate nutritional content but also in maintaining taste and texture when fortified with proteins. Protein fortification often alters the sensory and structural properties of baked goods, making it essential to identify suitable protein sources that balance nutrition and quality. Recent studies on hempseed-based baked goods, such as sponge cakes [21] and gluten-free bread [22], have demonstrated the potential of hempseed protein as an effective nutritional fortification alternative. Given its promising nutritional and functional benefits, hempseed protein isolate (HPI) can serve as a partial replacement for wheat flour to boost protein content while preserving product quality. One ideal application for this fortification is muffins, which are widely consumed as a breakfast and snack food. Muffin batter consists of a fat-in-water emulsion where egg, sugar, water, and fat form the continuous phase, with air bubbles as the discontinuous phase and flour particles dispersed throughout [23]. This emulsion structure is highly sensitive to changes in ingredient composition, making muffins an excellent model for studying the impact of protein fortification on quality characteristics [24].

The objective of this study was to evaluate the potential of hempseed protein isolate (HPI), derived from hemp cake, as a protein fortifier in vegan muffins, and to investigate the quality characteristics of muffins formulated with varying levels of HPI. This study explored the impact of HPI incorporation on muffin quality by analyzing the structural properties of both batter and baked muffins using rheometry, microscopy, texture analysis, and consumer sensory evaluation. Additionally, storage stability was assessed through textural and volume analysis over time. Therefore, this study aims to evaluate the potential of hempseed protein for protein fortification of vegan muffins and to provide a basis for further improvements in the quality of vegan bakeries.

## 2. Materials and Methods

### 2.1. Materials

Hempseed protein isolate (HPI), primarily sourced from a combination of hemp varieties grown in Canada and France, with 85% protein purity, containing <0.1% carbohydrates, 3.7% fat (0.6% saturated, 0.6% monounsaturated, 2.5% polyunsaturated, including 579.5 mg omega-3 and 1952 mg omega-6), 4.4% fiber, and trace amounts of salt, was obtained from Braham & Murray Good Hemp Co. (Barnstaple, Tewstock, England). The remaining ingredients, which included salt, sugar, baking soda, sunflower oil, lemon juice, almond milk, and wheat flour, were purchased from a local market situated in Daegu, Korea. Additionally, all chemical reagents required for the study were obtained from Duksan Chemicals (Ansan, Korea).

### 2.2. Preparation of Vegan Muffins

Vegan muffins were prepared following the formulation detailed in Table 1. HPI was incorporated at levels of 0%, 10%, 20%, and 30%, substituting wheat flour on a weight-to-weight (*w*/*w*) basis. Initially, sugar, baking soda, and salt were combined in a mixing bowl and blended thoroughly. Subsequently, buttermilk and sunflower oil were added to the mixer and mixed for 10 sec. The pre-prepared flour mixture, containing the specified HPI substitution levels, was then incorporated and mixed for 5 min at the second mixing stage to form a uniform batter. The batter was portioned into 50 g servings and deposited into baking pans (6.0 × 6.0 × 4.3 cm^3^). The muffins were baked in a preheated oven at 180 °C for 25 min.

### 2.3. Weight, Baking Loss, and Volume of Vegan Muffins

The weight of the muffins was measured 2 h after baking. Baking loss was determined by comparing the initial weight of the batter (W_1_) to the weight of the baked muffins after cooling (W_2_) [25]. The baking loss was calculated using the following Equation (1).

(1) $$Baking\;loss=((W_{1}-W_{2})÷W_{1})\times 100$$

The volume of the muffins was assessed using the rapeseed displacement method, following the procedure outlined in the AACC Method [26].

### 2.4. pH of Batter and Vegan Muffins

The pH of the muffin batter and crust was measured following the method described by Baek, Cho [27] with slight modifications. Briefly, 3 g of batter or muffin crust was combined with 27 mL of distilled water in a flask and homogenized for 1 min using a homogenizer (SH-001, Shimskyu, Tokyo, Japan). The homogenized mixture was centrifuged at 4000 rpm for 10 min (Labogene 1248R, Labogene Co., Ltd., Daejeon, Korea). The pH of the resulting supernatant was measured using a pH meter (FEP20, Mettler-Toledo, Schwerzenbach, Switzerland).

### 2.5. Color of Vegan Muffins

The color parameters (L*, a*, b*) of the muffin crust and crumb were measured using a Chroma Meter colorimeter (CR-300, Minolta Co., Osaka, Japan) in accordance with the CIE color measurement system. The color readings were expressed as Hunter values: L* for lightness, a* for redness, and b* for yellowness.

### 2.6. Viscosity of Muffin Batter

The viscosity of the muffin batter was measured using a viscometer (DV-II+ Pro, Brookfield, Middleboro, MA, USA) equipped with spindle No. 52. Measurements were conducted at 25 °C using 10 g of batter. Viscosity was recorded over a range of shear rates from 0 to 60 s^−1^. Each measurement was repeated three times to ensure accuracy. The resulting data were fitted to the Casson and Herschel–Bulkley models, described by the following equations:

(2) $$\sqrt{\tau }=\sqrt{\tau 0}+a\sqrt{\tau }$$

(3) $$\tau =K\gamma^{n}$$

where τ is shear stress (Pa), τ0 is yield stress (Pa), and γ is shear rate (s^−1^). The values of a and *K* are the consistency coefficient, and *n* is the flow behavior index.

### 2.7. Proximate Analysis of Vegan Muffins

Proximate analysis, including moisture, crude fat, protein (N × 6.25), and total ash content, was conducted using AOAC (2000) methods, while total carbohydrate content was determined using the phenol–sulfuric acid method [28].

### 2.8. Texture and Volume Changes of Vegan Muffins over Storage Period

Changes in texture and volume of the muffins were assessed at 2-day intervals over a 5-day storage period. The textural properties, including hardness, chewiness, cohesiveness, and springiness, were measured using a rheometer (Compac-100D, Sun Scientific Co., Tokyo, Japan). A double compression test was performed on the midsection of the muffin samples (10 × 10 × 10 mm^3^) to a depth of 30%, at a table speed of 60 mm/min and a maximum load of 2 kg. Each measurement was repeated six times to ensure consistency of results. The changes of volume of the muffins were determined using the rapeseed displacement method, with three replicates. The rate of volume reduction was calculated using the initial volume (V_0_) and the volume at a later time point (V_1_) according to the following formula:

(4) $$Volume\;reduction\;rate\left(\%\right)=\frac{V_{0}-V_{1}}{V_{0}}\times 100$$

### 2.9. Scanning Electron Microscope (SEM) of Vegan Muffins

A field emission scanning electron microscope (FE-SEM, SU8220, Hitachi, Mastsuda, Japan) was utilized to examine the cross-sectional microstructure of muffin crumb, following the method previously reported [29]. Muffin crumb samples were cut into cubes (4 mm × 4 mm × 4 mm) and immersed in a fixative solution containing 2.5% glutaraldehyde at 4 °C. After 24 h, the samples were rinsed in 0.1 M phosphate-buffered saline (PBS, pH 7.4) for 15 min at 60 rpm to remove excess fixative. The samples then underwent a graded ethanol dehydration process using sequential concentrations of 30%, 50%, 70%, 90%, and 100% ethanol. Ethanol solutions were replaced every 15 min to ensure complete dehydration. The dehydrated samples were dried in an oven at 35 °C for 48 h. Once fully dried, the specimens were mounted on aluminum stubs with carbon paste and coated with platinum using a sputter coater. Microstructural imaging was conducted at an accelerating voltage of 10 kV.

### 2.10. Fourier Transform Infrared (FT-IR) Spectroscopy of Vegan Muffins

FT-IR spectroscopy was employed to investigate the chemical structure of vegan muffins with HPI. The muffin samples were ground and mixed with potassium bromide (KBr) at a 1:200 ratio, then applied to an FT-IR spectrophotometer (Frontier, PerkinElmer, Hopkinton, MA, USA) with a resolution of 4 cm^−1^ across a frequency range of 4000 to 400 cm^−1^ [30].

### 2.11. Antioxidant Activity of Vegan Muffins

The total polyphenol content (TPC) of HPI was determined using a modified Folin–Ciocalteu method as described by Vl [31]. The procedure involved combining 0.1 mL of sample with 0.05 mL of Folin–Ciocalteu reagent, followed by the addition of 0.3 mL of 2% sodium carbonate (Na_2_CO_3_) solution. After a 15 min incubation at room temperature for color development, 1 mL of distilled water was added to the reaction mixture. The absorbance was then measured at 725 nm using a Shimadzu UV-2550 UV–Visible spectrophotometer (Shimadzu Corporation, Tokyo, Japan). TPC concentration (mg GAE/g) was quantified by referencing a standard calibration curve prepared with gallic acid.

The antioxidant activity of the muffins was evaluated using the ABTS radical scavenging assay. ABTS free radicals were generated by reacting 7 mM ABTS with 2.45 mM potassium persulfate for 12–16 h at room temperature in the dark. Muffin crumbs were defatted by refluxing in hexane (1:20 *w*/*v*) for 1 h, and the residue was dried at 40 °C for 6 h before analysis. The defatted muffin samples were then mixed with 70% ethanol at four different concentrations (0.025, 0.05, 0.1, and 0.2 g/mL). Bioactive compounds were extracted by incubating the mixtures at 20 °C for 20 h in a shaking incubator (SI-600R, JeioTech, Daejeon, Korea) at 200 rpm. The mixtures were subsequently centrifuged at 4000 rpm for 10 min (SUPRA-22K centrifuge, Hanil Science Industrial, Incheon, Republic of Korea). A 50 µL aliquot of the supernatant was added to 950 µL of the ABTS solution and allowed to react in the dark for 30 min. Absorbance was measured at 734 nm using ethanol as the blank. The ABTS radical scavenging activity (%) was calculated using the following equation:

(5) $$Antioxidant\;activity\left(\%\right)=(\left(A_{0}-A_{1}\right)÷A_{0})\times 100$$

where A_1_ and A_0_ are the absorbance of the sample and blank, respectively.

### 2.12. In Vitro Protein Digestibility (IVPD) of Vegan Muffins

The in vitro protein digestibility (IVPD) of the muffins was assessed using the pepsin and pancreatin digestion method described by Cho, Olawuyi [25]. The total protein content of the muffins, both before and after the two-stage digestion process, was quantified using the Lowry protein assay [32]. IVPD was calculated by subtracting the undigested protein content from the initial total protein content of the sample, using the following equation:

(6) $$IVPD\left(\%\right)=((Initial\;protein-Final\;undigested\;protein)÷Initial\;protein)\times 100$$

### 2.13. Sensory Evaluation of Vegan Muffins

Sensory evaluation of the vegan muffins containing HPI was conducted with a panel of 30 semi-trained assessors [33,34], comprising undergraduate and graduate students from the Department of Food Science and Technology, Kyungpook National University (KNU), Republic of Korea, all of whom were over 19 years of age. The evaluation took place 3 h after baking, assessing sensory attributes such as appearance, aroma, taste, mouthfeel, and overall acceptability. Muffin samples were cut into 30 × 30 × 30 mm cubes. Two pieces of crumb from each sample were placed on plastic plates labeled with random three-digit codes and presented to the panelists in a randomized order. The evaluation was conducted in an air-conditioned room at 25 °C under white light, with water provided for palate cleansing between samples. Panelists rated each attribute using a seven-point hedonic scale (1 = Dislike very much, 2 = Dislike moderately, 3 = Dislike slightly, 4 = Neither like nor dislike, 5 = Like slightly, 6 = Like moderately, 7 = Like very much). The sensory evaluation was approved by the Research Ethics Committee at Kyungpook National University (Approval No. 2024–0397). The panelists were trained according to ISO 8586:2012 and were thoroughly briefed on the study’s objectives and evaluation procedures before providing informed consent [35].

### 2.14. Statistical Analysis

All experiments were conducted in triplicate, and the results are presented in tables and graphs as the mean ± standard deviation. Statistical analysis was performed using Duncan’s multiple range test to compare means at the 95% significance level (*p* < 0.05). The analysis was carried out using SPSS software, version 22 (SPSS Inc., Chicago, IL, USA). Duncan’s Multiple Range Test was used for post hoc analysis due to its sensitivity in detecting significant differences between group means after ANOVA, making it suitable for comparing the effects of varying HPI concentrations on muffin properties.

## 3. Results and Discussion

### 3.1. Physical and Baking Properties of Vegan Muffins

The results for weight and baking loss are presented in Table 2. The weight of the baked muffins ranged from 39.47 to 40.12 g, while baking loss ranged from 19.77% to 21.33%. A positive correlation was observed between HPI content and muffin weight, whereas baking loss showed an inverse relationship. Muffins with the highest HPI substitution (HP30) exhibited the highest weight and the lowest baking loss, while control muffins (CON) had the lowest weight and the highest baking loss. These trends can be attributed to the high water-holding capacity (WHC) of HPI, which reduces the amount of free water in the batter, thereby limiting moisture loss during baking [36]. Additionally, increased protein content promotes hydration and gelation, further entrapping water within the muffin matrix. This phenomenon aligns with findings from previous studies [37], which reported high water absorption when whey and soy proteins were added to wheat flour bread. The protein network formed by HPI stabilizes the batter structure, reducing collapse and minimizing moisture loss. Furthermore, the thermal properties of the batter may be altered by HPI, leading to quicker crust formation and reduced moisture evaporation.

As shown in Table 2, the volume of muffins with HPI addition is significantly larger than that of the control (CON) muffins, and muffin volume increases proportionally with the amount of HPI. This can be attributed to the significant foaming capacity of HPI [38], as evidenced by the bubble structure observed in the muffin cross-sections (Figure 3). The superior foaming and emulsifying properties of HPI enhance the stability of air bubbles, resulting in a more aerated structure [39]. Additionally, during baking, HPI undergoes thermal denaturation, leading to the formation of an inter-protein network that supports volume expansion [40]. These properties make HPI highly advantageous for baking applications, improving the quality and structural integrity of baked products.

### 3.2. pH of Batter and Vegan Muffins

The pH values of both the muffin batter and crust exhibit a clear trend with increasing HPI content (Table 2). The batter’s pH decreases progressively, ranging from 8.05 to 6.23, as more HPI is incorporated. This reduction is primarily attributed to the amino acid profile of HPI, which is rich in acidic amino acids such as glutamic acid, arginine, and aspartic acid. These amino acids can dissociate and release hydrogen ions (H^+^) in the batter, contributing to a more acidic environment [41]. Furthermore, the amino acids in hemp protein can act as buffers, maintaining a slightly acidic pH as more HPI is added to the batter. During mixing and the initial stages of baking, some proteins may partially denature, exposing more acidic groups and further contributing to pH reduction. In baked muffins, the pH follows a similar decreasing trend with higher HPI content but remains slightly higher than the batter, ranging from 8.38 to 6.27. This slight increase in pH during baking is likely due to the reaction of baking soda with acidic components, producing basic substances like Na_2_CO_3_ [42]. Furthermore, the heat applied during baking may alter protein structures and interactions, potentially influencing the lower pH of the muffins [43].

### 3.3. Color of Vegan Muffins

The color analysis of the muffin crust and crumb, measured using the CIE Lab color space (Table 3), reveals significant changes with the addition of HPI. Lightness (L) values for the crust decrease from 66.49 to 47.43 as HPI content increases, likely due to the inherent brownish color of HPI, which darkens the muffins with higher protein addition. In contrast, redness (a*) values consistently increase from HP10 to HP30 in both the crust and crumb, suggesting that higher HPI levels contribute to a more pronounced reddish hue. This increase in redness can be attributed to the Maillard reaction, where reducing sugars and amino acids interact during baking to form reddish-brown pigments [44]. The specific amino acid composition of HPI may enhance this reaction, leading to deeper red tones with higher protein incorporation. Although the Maillard reaction intensifies with higher protein content due to the increased availability of amino acids, the reaction products tend to favor the formation of darker, brownish, and reddish pigments rather than yellow hues. Consequently, as HPI content increases, the formation of reddish-brown compounds reduces the intensity of yellowness (b*). Similar to this study, wheat bread fortified with 20% and 30% *Phaseolus coccineus* seed flours exhibited lower lightness (L), higher redness (a), and lower yellowness (b*) in crust samples compared to the control [45], reflecting the impact of protein-rich ingredients on enhancing Maillard reaction products and modifying color.

### 3.4. Viscosity of the Batter

**Figure 1:**
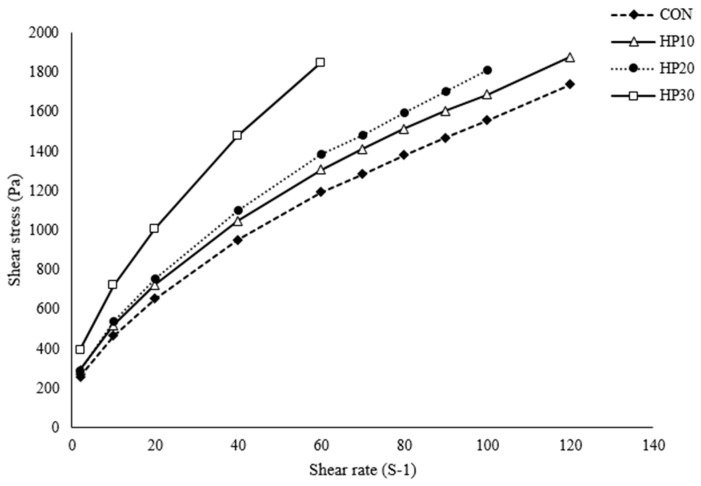
Viscosity of the batter.

The shear stress of the muffin batter, as illustrated in Figure 1, demonstrates a clear increase with rising shear rate, exhibiting typical shear-thinning behavior. Batters fortified with higher levels of HPIs displayed significantly greater shear stress compared to the control (CON), with the curves steepening as HPI content increased, indicating that higher HPI content enhances the batter’s resistance to flow. The experimental data were accurately modeled using the Casson and Herschel–Bulkley equations (Table 4), with R^2^ values ranging from 0.995 to 0.999, confirming a strong fit. Yield stress values also increased with HPI content, suggesting that HPI strengthens the batter structure primarily through enhanced protein interactions and network formation. During batter formation, proteins in the HPI interact both with each other and with gluten from the wheat flour, forming a robust three-dimensional network [46]. This network effectively traps moisture and enhances the structural integrity of the batter, thereby increasing its resistance to deformation. Additionally, HPI’s high water-holding capacity allows it to absorb and retain moisture effectively; by binding more water, HPI reduces the availability of free water in the batter, which further increases viscosity and shear stress measurements. These findings are consistent with previous reports indicating that protein-rich doughs exhibit higher viscosity and yield stress [47]. Moreover, as HPI hydrates during mixing, it forms a gel-like structure that thickens the batter due to protein unfolding and interaction with water molecules.

The flow behavior index (n) derived from the Herschel–Bulkley model indicates that all muffin batter samples exhibit shear-thinning (pseudoplastic; n < 1) behavior [48], with n values ranging from 0.59 to 0.66 (Table 4). This shear-thinning behavior reflects a decrease in batter viscosity as the shear rate increases, facilitating easier flow under higher stress conditions. The consistency coefficient (K), which reflects the resistance to fluid flow [49], increases with higher HPI content, indicating reduced fluidity and greater internal resistance within the batter. This trend suggests that HPI fortification enhances viscosity due to its ability to bind water and form a more robust protein network. Additionally, the increase in the flow behavior index (*a*) with rising HPI levels points to a more pronounced shear-thinning response associated with higher protein content. These results confirm that the muffin batters are non-Newtonian fluids, maintaining higher viscosity at low shear rates while becoming less viscous at higher shear rates. Such rheological properties are advantageous during mixing and processing, as they facilitate batter flow while ensuring structural integrity and stability in the final baked product.

### 3.5. Proximate Analysis of Vegan Muffins

The proximate composition of the muffins is presented in Table 5. The increase in protein content observed in HPI-fortified muffins ranges from 1.4-fold (HP10) to 2-fold (HP30) compared to the control (CON). Although HPI has a high initial protein content of 92%, its partial substitution for wheat flour at levels of 10%, 20%, and 30% means that only a fraction of the total flour is replaced with this high-protein ingredient. Consequently, the remaining wheat flour and other components, such as carbohydrates, fats, and moisture, dilute the overall protein contribution. The control muffins contain 9.61% protein, primarily from wheat flour, while HPI fortification increases protein levels to 13.27% (HP10), 17.16% (HP20), and 19.40% (HP30). The actual protein content was expected to be higher in vegan muffins fortified with HPIs, however, during baking, heat causes protein denaturation, breaking bonds in the protein structure and exposing amino acids [50]. Additionally, these amino acids participate in the Maillard reaction with reducing sugars, forming complex browning compounds [51]. The carbohydrate content of the muffins ranged from 17.66% to 25.43%, decreasing as the HPI substitution level increased. This reduction is due to the partial replacement of wheat flour, a primary source of carbohydrates. Moisture is an important factor in the shelf life, texture, and overall quality of baked products by interacting directly or indirectly with other food ingredients [52]. The moisture content of the muffins increased with higher levels of protein addition, ranging from 40.24% in the CON to 42.04% in HP30. This increase can be attributed to the high WHC of HPI, which binds water in the batter and retains it during baking. The WHC of HPI arises from its hydrophilic groups and the protein denaturation process, which exposes additional water-binding sites. HPI also interferes with gluten network formation and competes with starch for water during gelatinization, further enhancing moisture retention. This mechanism is reflected in the baking loss results, where muffins with higher HPI content exhibited lower moisture loss. The increased moisture content helps delay muffin aging, reduce texture stiffness, and improve the overall product quality and freshness [53]. No significant difference in fat content was observed among the muffins, likely due to the consistent amount of oil used in all formulations. The ash content of the muffins ranged from 0.50% to 0.92%. The control (CON) muffins had an ash content of 0.50%, consistent with the mineral content typically found in pure wheat flour. Ash content serves as an indicator of the mineral concentration in a product [54,55]. The increase in ash content with higher levels of HPI can be attributed to the naturally higher mineral content in HPI, which contributes to the overall ash content of the fortified muffins.

### 3.6. Texture and Volume Change of Vegan Muffins over the Storage Period

**Figure 2:**
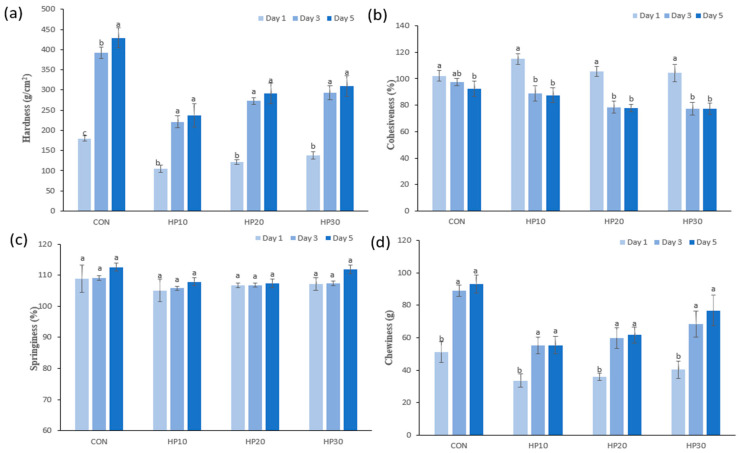
Texture change of muffins (**a**) hardness, (**b**) cohesiveness, (**c**) springiness, and (**d**) chewiness during the storage period. Different superscript letters (^a–c^) show significant differences (*p* < 0.05) within the column.

TPA was used to evaluate parameters such as springiness, chewiness, cohesiveness, and hardness, providing insights into the muffins’ structural behavior during consumption. Measurements were taken on the first and fifth days after baking to assess changes over time. Hardness describes the force required to deform the food in the mouth [56], with the results demonstrated in Figure 2a. On the first day, the control muffins exhibited the highest hardness (179.72 g/cm^2^), while HPI-fortified muffins had lower values, ranging from 104.89 g/cm^2^ (HP10) to 137.73 g/cm^2^ (HP30). The initial reduction in hardness with HPI addition is attributed to its high water-holding capacity, which increases moisture retention and softens the muffins by reducing the density of the protein–starch matrix. This trend aligns with findings by Shevkani and Singh [57] who observed decreased hardness in gluten-free muffins fortified with pea and amaranth proteins. Interestingly, HP30 exhibited higher hardness compared to HP10, likely due to increased protein content and heat-induced protein denaturation, which promotes stronger bonds between amino acids and protein coagulation [58,59]. As expected, hardness increased across all samples over five days, reflecting the onset of staling. However, HPI-fortified muffins consistently displayed lower hardness values (236.84–309.88 g/cm^2^) compared to the control (429.25 g/cm^2^). This suggests that HPI fortification delays staling, likely due to higher moisture retention and protein–gluten interactions that slow moisture migration and starch retrogradation, processes responsible for texture firming during storage. Cohesiveness, which reflects the internal bonding strength that maintains the food’s structure [27], decreased across all muffin samples over the 5-day storage period (Figure 2b). On Day 1, the CON exhibited a cohesiveness of 102.25%, while HPI-fortified samples showed higher values, with HP10 having the highest cohesiveness (114.87%), followed by HP20 (105.50%) and HP30 (104.29%). However, as storage progressed, cohesiveness significantly decreased, particularly in the HPI-fortified muffins. By Day 5, HP10 dropped to 87.52%, while HP20 and HP30 reached 77.84% and 77.29%, respectively, compared to the control (92.52%). This decline aligns with findings by Çabuk [60] and Bruttomesso, Bianchi [61] who observed decreased cohesiveness in protein-enriched baked goods due to gluten weakening caused by flour substitution [62]. Springiness, which measures the ability of muffins to return to their original shape after deformation [63], remained relatively stable across all samples (Figure 2c). On Day 1, CON exhibited the highest springiness (108.82%), while HPI-fortified muffins showed slightly lower values, ranging from 105.07% (HP10) to 107.14% (HP30). By Day 5, springiness increased slightly in all samples, with the control reaching 112.58% and HP30 showing a comparable value (111.88%). The smaller changes in HP10 and HP20 suggest that moderate HPI levels stabilize muffin elasticity, while higher levels (HP30) maintain structural integrity, likely due to HPI’s water-holding capacity, which preserves the muffin matrix [64]. Chewiness, representing the energy required to chew food for swallowing, showed greater variation over time [65] (Figure 2d). On Day 1, the control muffins had the highest chewiness (51.09 g), while HPI-fortified samples exhibited lower values: 33.62 g (HP10), 35.96 g (HP20), and 40.32 g (HP30). This initial reduction can be attributed to HPI’s ability to retain moisture, softening the muffin structure. However, chewiness increased across all samples during storage, reflecting texture firming. By Day 5, chewiness in the control muffins reached 92.96 g, significantly higher than in HPI-fortified muffins. HP10 and HP20 displayed slower increases, reaching 55.41 g and 61.65 g, respectively, while HP30 exhibited the highest chewiness among fortified samples (76.69 g). The increase in chewiness with higher HPI levels (HP30) may be due to the formation of a denser protein matrix over time, which enhances resistance to deformation. These data align with the findings of Rouzbahani, Hosseini [66], who observed increased chewiness and hardness with higher levels of mycoprotein addition in protein-fortified sponge cakes.

The volume change of muffins over the 5-day storage period (Table 6) shows clear trends influenced by HPI fortification. From Day 1 to Day 3, the control (CON) exhibited the smallest volume change (2.92 ± 1.00%), indicating greater structural stability due to the intact gluten network. In contrast, HPI-fortified samples showed larger volume changes: HP10 (4.22 ± 0.81%), HP20 (4.07 ± 1.75%), and HP30 (4.13 ± 1.37%), likely due to increased moisture retention disrupting the gluten matrix. Between Day 3 and Day 5, the control maintained moderate volume change (3.33 ± 0.40%), while HP10 and HP20 displayed similar changes (4.35 ± 1.38% and 4.12 ± 0.24%). However, HP30 showed the highest volume change (6.46 ± 1.38%), suggesting that higher HPI levels lead to greater structural expansion due to excess moisture retention and gluten weakening. These volume changes are primarily due to moisture evaporation during storage. Muffins with higher HPI content retain more water, leading to more significant volume loss as moisture evaporates until equilibrium with the surrounding environment is reached [67]. Interestingly, while the control had the smallest volume change, it exhibited the highest texture change, likely because its moisture content equilibrating faster due to lower water retention. This indicates that higher volume loss in HPI-fortified samples does not directly correlate with texture changes.

### 3.7. Scanning Electron Microscopy of Vegan Muffins

**Figure 3:**
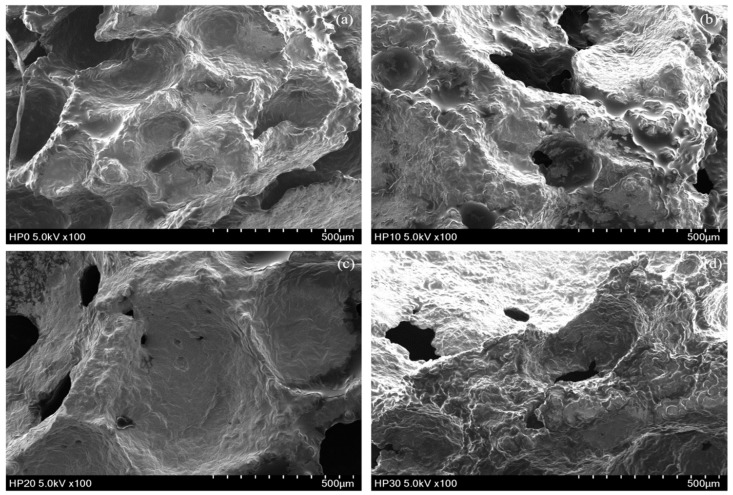
Microstructure analysis of vegan muffin crumbs. (**a**) CON, (**b**) HP10, (**c**) HP20, (**d**) HP30.

The structural analysis of muffin crumbs (Figure 3) reveals distinct differences in microstructure that correlate well with the texture analysis results. The CON exhibited a dense and tightly packed crumb structure with minimal visible pores. This compact microstructure corresponds to the highest hardness and chewiness values observed, as the lack of air pockets increases crumb density, making it more resistant to deformation and more difficult to chew. In contrast, HP10 displayed a highly porous structure with large air pockets, resulting in significantly lower hardness and chewiness. The increased porosity reduces crumb density, making the muffin softer and more compressible, with lower resistance to deformation and easier chewing, consistent with studies linking higher porosity to softer muffin textures [68]. The microstructure of HP20 showed a balance between compactness and porosity, with smaller but still visible pores compared to HP10. This moderately dense structure aligns with the intermediate hardness and chewiness values, indicating a texture that is firmer than HP10 but softer than CON. HP30 exhibited a dense structure with very small and sparse pores, similar to the control but with slightly more porosity. This crumb structure corresponds to the highest hardness and chewiness among the HPI-fortified muffins, though still lower than the control. The reduced porosity in HP30 results in a denser crumb, contributing to a firmer texture and increased resistance to chewing. Higher levels of protein incorporation have been noted to cause disruption of gluten formation and reduced ability to retain carbon dioxide during leavening, as noted by Franco-Miranda et al. [58], leading to smaller and more numerous alveoli. This results in a crumb with fewer and less uniform air pockets, making the structure more compact. The inability to form a well-developed gluten network means that the dough cannot effectively trap gas, leading to reduced aeration and more tightly packed crumb components. In addition, protein fortification increases water absorption in the dough, limiting water availability for gluten hydration [69]. This competition impairs gluten network formation, leading to a denser crumb structure. Hence, these structural changes directly influenced texture, with denser crumbs (CON and HP30) resulting in higher hardness and chewiness, while more porous crumbs (HP10 and HP20) produced softer textures and lower chewiness.

### 3.8. FT-IR Analysis of Vegan Muffins

**Figure 4:**
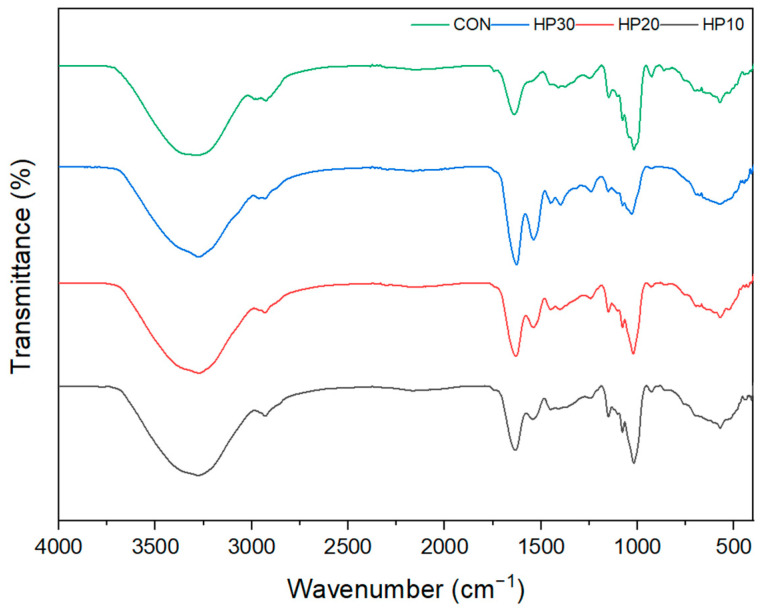
FT-IR analysis of vegan muffin crumbs.

The FTIR spectra of muffins reveal distinct trends with increasing HPI fortification, particularly in the Amide I, Amide II, and Amide III bands, reflecting changes in protein structure and interactions within the muffin matrix (Figure 4). CON shows a band at 3284 cm^−1^, while the HPI-fortified muffins display bands at 3271–3275 cm^−1^, corresponding to the O–H stretching vibration of wheat flour or the N–H stretching vibration of protein [70]. This shift suggests enhanced hydrogen bonding and higher water retention due to the water-holding capacity of HPI. The presence of HPI increases protein–water interactions, leading to greater moisture retention in the muffin matrix. In the Amide I region (1700–1600 cm^−1^), peaks shift to lower wavenumbers (red shift) with increasing HPI content: 1639 cm^−1^ for the control (CON), 1633 cm^−1^ for HP10, 1631 cm^−1^ for HP20, and 1628 cm^−1^ for HP30. These peaks correspond to the C=O stretching vibrations of proteins [71]. The red shift indicates alterations in the secondary structure of proteins, likely due to increased hydrogen bonding and the formation of β-sheet structures caused by interactions between HPI proteins and the gluten network. The higher protein content from HPI fortification promotes these structural changes during baking. The Amide II region (1580–1510 cm^−1^) also shows a red shift, with peaks at 1542 cm^−1^ for HP10 and 1539 cm^−1^ for both HP20 and HP30. These peaks are associated with N–H bending and C–N stretching vibrations [72], reflecting the increasing protein content and the disruption of the gluten network as more HPI is incorporated. The consistent peak positions for HP20 and HP30 suggest a saturation point in the protein network where further HPI addition does not significantly alter the structure. In the Amide III region (1300–1200 cm^−1^), peaks shift from 1249 cm^−1^ in the control to 1245 cm^−1^ in HP10, 1243 cm^−1^ in HP20, and 1242 cm^−1^ in HP30. This region, associated with C–N stretching and N–H bending, reflects protein denaturation and the formation of new protein interactions during baking. The red shift indicates structural reorganization due to increased HPI content, likely driven by additional hydrogen bonding and protein conformational changes. The band near 2962–2929 cm^−1^, attributed to C–H stretching of methylene groups, appears consistently across all samples, indicating the presence of lipid components [70]. Additionally, bands at 1150–1151 cm^−1^ and 1018–1022 cm^−1^ reflect C–H and C–O stretching vibrations in the muffin matrix, suggesting interactions between proteins and other components of the batter, such as gluten and lipids. These structural changes observed in the Amide I, II, and III bands correlate with the increased hardness and chewiness in HPI-fortified muffins. The incorporation of HPI disrupts the gluten network, leading to the formation of a denser protein matrix and enhanced protein–protein interactions. This corresponds with the observed textural changes, where higher HPI levels influence moisture retention and contribute to firmer textures.

### 3.9. Antioxidant Activity of Vegan Muffins

**Figure 5:**
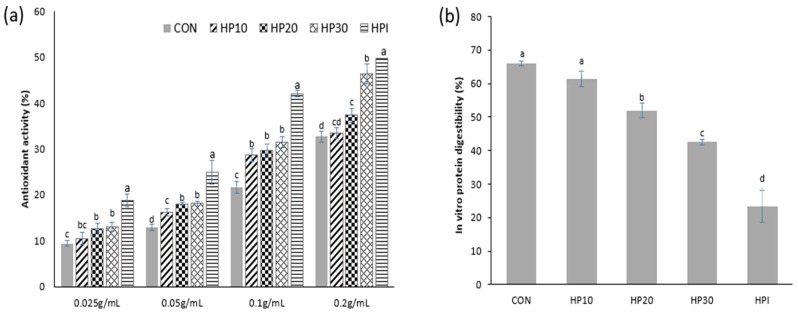
(**a**) ABTS radical activity (%) and (**b**) IVPD (%) of muffins. Different superscript letters (^a–d^) show significant differences (*p* < 0.05) within the column.

The antioxidant activity of muffins was assessed using the ABTS radical scavenging assay, as presented in Figure 5a. The highest antioxidant activity was observed in HP30 (13.06–46.28%), followed by HP20 (12.64–37.33%) and HP10 (10.50–33.40%). The control sample exhibited the lowest antioxidant activity, ranging from 9.42 to 32.66%. The antioxidant activity increased with higher HPI fortification due to the antioxidant peptides in HPI, which donate electrons and hydrogen atoms to neutralize free radicals. Higher HPI concentrations enhance peptide availability, boosting radical scavenging activity [15]. This result is further supported by the high total phenolic content (TPC) in HPI, measured at 144.67 mg GAE/g. In comparison, previous studies have reported that dehulled hempseed exhibits a higher TPC (193.33 mg FAE/100 g), while whole hempseed and hempseed cake contain even higher levels at 384.50 mg FAE/100 g and 247.45 mg FAE/100 g, respectively, highlighting the rich phenolic profile of hemp-derived products [73]. Although the extracted HPI in this study showed a lower TPC than other hemp-derived products, it still contributed significantly to the antioxidant potential of the fortified muffins. Additionally, prior research has established that the antioxidant capacity of proteins is closely linked to the bioactive peptides formed during hydrolysis [74,75], suggesting potential use in functional food and nutraceutical sectors.

### 3.10. IVPD of Vegan Muffins

The IVPD measures the proportion of proteins broken down by digestive enzymes and absorbed as amino acids or other nitrogen compounds, reflecting the efficiency of protein utilization [25]. It serves as a nutritional metric to assess how effectively a protein source is used by the body; higher IVPD values indicate greater amino acid absorption and utilization [76]. In this study, the IVPD of the muffins ranged from 42.63% to 66.08% (Figure 5b). The control samples exhibited the highest IVPD (66.08%), while IVPD decreased with increasing HPI fortification. Notably, pure HPI displayed the lowest IVPD among all samples (23.41%). This reduction in digestibility may be due to the higher protein content and structural changes in fortified muffins, making proteins more difficult to digest. Similar trends have been reported in previous studies, where interactions with starch, fiber, and lipids inhibited protein hydrolysis [77]. The study by Ruiz, Xiao [78] reported lower digestibility in whole quinoa flour compared to quinoa protein extract due to these inhibitory effects. These findings suggest that in HPI-fortified muffins, similar interactions between proteins, starch, and lipids may contribute to the reduced digestibility observed with higher HPI levels.

### 3.11. Sensory Evaluation of Vegan Muffins

The sensory evaluation results for appearance, aroma, taste, mouthfeel, and overall acceptability of muffins with HPI fortification are summarized in Table 7. The control (CON) scored the highest for appearance (5.77), while HP10 had the lowest score (4.07). The decrease in appearance with HPI substitution is likely due to the altered color caused by the distinctive pigments in HPI, as observed in Table 3 and reported in previous studies involving protein-rich flour substitutes [79]. However, appearance scores improved with higher substitution levels, suggesting that the deeper hue from higher HPI content may enhance visual appeal. Aroma scores showed no substantial differences among samples, ranging from 4.73 to 5.27. This uniformity implies that HPI substitution does not significantly impact the muffins’ olfactory profile, likely due to the mild aroma of hempseed. Similarly, taste scores were consistent across all formulations, ranging from 4.90 to 5.10, indicating moderate acceptance. This suggests that replacing up to 30% of wheat flour with HPI does not alter the flavor, as HPI’s subtle, nutty taste blends well with the muffin matrix without overpowering the overall flavor profile [80]. These findings align with prior research on hempseed oil press-cake used in gluten-free crackers [81]. Mouthfeel ratings varied slightly, ranging from 4.83 to 5.43, indicating that the combination of HPI and wheat flour maintains a balanced texture. The structural properties contributed by HPI appear to integrate well with wheat flour, preserving a pleasant mouthfeel without significant changes in texture. For overall acceptability, scores ranged between 4.83 and 5.33, showing no significant differences among the samples. This indicates that HPI fortification up to 30% is generally well-accepted by consumers, achieving overall acceptability similar to the control wheat flour-based muffins [82]. Overall, there were no significant differences in all attributes except appearance, indicating a high potential for food applications of HPI as protein fortification in muffins.

## 4. Conclusions

This study demonstrates the successful development of vegan protein-fortified muffins through the partial replacement of wheat flour with hempseed protein isolate (HPI), derived from the upcycling of hemp meal. The incorporation of HPI (10–30%) led to significant improvements in the muffins’ nutritional, functional, and structural properties, addressing the growing demand for protein-rich, sustainable, plant-based baked goods. Rheological analysis indicated that higher HPI inclusion significantly increased moisture content and reduced baking loss, resulting in a softer dough network with greater capacity for dough expansion during baking. This was supported by scanning electron microscopy (SEM), which revealed a more aerated crumb structure with larger and more evenly distributed air pores, especially at 10% HPI substitution. Batter viscosity also increased with HPI fortification, suggesting enhanced resistance to deformation and flow due to stronger protein–protein interactions. Texture profile analysis (TPA) showed that muffins fortified with 20% and 30% HPI exhibited higher hardness and reduced elasticity during storage compared to those with 10% HPI over the storage period. These changes correlated with FTIR spectroscopy findings, which revealed redshifts in the Amide I and II bands, indicating modifications in protein secondary structures and stronger protein–protein and protein–gluten interactions. HPI fortification also enhanced the muffins’ antioxidant activity, with ABTS radical scavenging values increasing from 32.66% (CON) to 46.28% (HP30), attributed to the bioactive peptides and phenolic compounds present in HPI. However, the in vitro protein digestibility (IVPD) decreased from 66.08% (CON) to 42.63% (HP30), likely due to interactions between proteins, starch, and lipids that inhibited enzymatic hydrolysis. Sensory evaluation revealed no significant differences in aroma, taste, mouthfeel, or overall acceptability, with scores ranging from 4.83 to 5.43. Among all formulations, HP30 (30% HPI substitution) emerged as the optimal level, offering the best balance of high protein content, improved texture, enhanced antioxidant activity, and acceptable sensory quality. These findings highlight HPI’s potential as a sustainable, upcycled protein fortifier that improves the nutritional, functional, and antioxidant properties of vegan muffins while maintaining consumer acceptance. Future research should focus on optimizing HPI processing methods (e.g., thermal treatments) to further improve digestibility, sensory quality, and shelf-life stability of HPI-fortified products, aligning with consumer demands for healthier, protein-enriched, and sustainable vegan alternatives.

## Figures and Tables

**Table 1:** Composition of vegan muffin.

Ingredient (g)	CON	HP10	HP20	HP30
Hemp protein isolate	0	7	14	21
Wheat flour	70	63	56	49
Sugar	24	24	24	24
Baking soda	1	1	1	1
Sunflower oil	24	24	24	24
Butter milk	70	70	70	70
Salt	0.4	0.4	0.4	0.4

CON: muffin with 0% wheat flour replaced by HPI; HP10: muffin with 10% wheat flour replaced by HPI; HP20: muffin with 20% wheat flour replaced by HPI; HP30: muffin with 30% wheat flour replaced by HPI.

**Table 2:** Baking properties and pH of vegan muffins.

Samples	Weight (g)	Volume (mL)	Baking Loss (%)	pH	
				Batter	Muffin
CON	39.47 ± 0.26 ^a^	63.33 ± 3.33 ^b^	21.33 ± 1.16 ^a^	8.05 ± 0.03 ^a^	9.38 ± 0.01 ^a^
HP10	40.12 ± 0.55 ^a^	76.50 ± 2.78 ^a^	20.86 ± 1.36 ^b^	7.20 ± 0.00 ^b^	7.66 ± 0.01 ^b^
HP20	40.19 ± 0.53 ^a^	81.50 ± 5.27 ^a^	20.22 ± 0.82 ^b^	6.57 ± 0.00 ^c^	6.67 ± 0.05 ^c^
HP30	40.49 ± 0.33 ^a^	81.83 ± 4.48 ^a^	19.77 ± 0.57 ^b^	6.23 ± 0.01 ^d^	6.19 ± 0.01 ^d^

Different superscript letters (^a–d^) show significant differences (*p* < 0.05) within the column.

**Table 3:** Hunter’s color values of vegan muffins.

Samples	Crust			Crumb		
	L*	a*	b*	L*	a*	b*
CON	66.49 ± 1.45 ^a^	0.52 ± 0.33 ^b^	30.48 ± 1.21 ^a^	69.78 ± 0.29 ^a^	−2.88 ± 0.04 ^c^	18.51 ± 0.33 ^a^
HP10	60.69 ± 1.58 ^b^	0.73 ± 0.38 ^b^	20.18 ± 1.27 ^b^	61.11 ± 1.71 ^b^	0.36 ± 0.09 ^b^	14.45 ± 0.35 ^b^
HP20	54.74 ± 0.88 ^c^	1.53 ± 0.20 ^a^	20.03 ± 0.98 ^b^	53.76 ± 2.20 ^c^	0.48 ± 0.15 ^b^	14.32 ± 0.40 ^b^
HP30	47.43 ± 2.11 ^d^	1.98 ± 0.30 ^a^	18.77 ± 1.26 ^b^	48.17 ± 1.40 ^d^	0.91 ± 0.28 ^a^	14.08 ± 1.04 ^b^

Different superscript letters (^a–d^) show significant differences (*p* < 0.05) within the column.

**Table 4:** The Casson and Herschel–Bulkley parameters of batter.

Samples	Casson			Herschel–Bulkley		
	Yield Stress (Pa)	*a*	R^2^	*K*	*n*	R^2^
CON	174.50	2.67	0.996	86.78	0.61	0.999
HP10	203.10	2.74	0.995	104.95	0.59	0.999
HP20	190.12	2.94	0.996	105.75	0.60	0.999
HP30	228.42	3.66	0.999	110.47	0.66	0.999

*a*, *K*: consistency coefficient, *n*: flow behavior index, R^2^: coefficient of determination.

**Table 5:** Proximate composition of vegan muffins.

Samples	Protein (%)	Carbohydrate (%)	Moisture (%)	Fat (%)	Ash (%)
CON	9.61 ± 0.19 ^d^	25.43 ± 0.80 ^a^	40.24 ± 2.38 ^a^	10.14 ± 1.04 ^a^	0.50 ± 0.02 ^c^
HP10	13.27 ± 0.54 ^c^	22.73 ± 1.04 ^b^	41.42 ± 1.19 ^a^	10.82 ± 0.77 ^a^	0.56 ± 0.06 ^bc^
HP20	17.16 ± 0.11 ^b^	19.36 ± 0.19 ^c^	41.70 ± 0.44 ^a^	11.14 ± 1.04 ^a^	0.72 ± 0.10 ^b^
HP30	19.40 ± 0.51 ^a^	17.66 ± 0.09 ^c^	42.04 ± 0.20 ^a^	11.16 ± 0.77 ^a^	0.92 ± 0.08 ^a^

Different superscript letters (^a–d^) show significant differences (*p* < 0.05) within the column.

**Table 6:** Volume change of muffins during the storage period.

	Volume Change (%) (Day 1–3)	Volume Change (%) (Day 3–5)
CON	2.92 ± 1.00 ^a^	3.33 ± 0.40 ^b^
HP10	4.22 ± 0.81 ^a^	4.35 ± 1.38 ^ab^
HP20	4.07 ± 1.75 ^a^	4.12 ± 0.24 ^ab^
HP30	4.13 ± 1.37 ^a^	6.46 ± 1.38 ^a^

Different superscript letters (^a,b^) show significant differences (*p* < 0.05) within the column.

**Table 7:** Sensory evaluation of muffins.

	Appearance	Aroma	Taste	Mouthfeel	Overall Acceptability
CON	5.77 ± 1.38 ^a^	5.27 ± 1.14 ^a^	5.10 ± 1.24 ^a^	5.20 ± 1.42 ^a^	5.33 ± 1.42 ^a^
HP10	4.07 ± 1.44 ^c^	5.13 ± 1.04 ^a^	5.10 ± 1.24 ^a^	5.43 ± 1.10 ^a^	5.00 ± 1.39 ^a^
HP20	4.67 ± 1.03 ^bc^	4.73 ± 1.01 ^a^	5.10 ± 1.24 ^a^	5.23 ± 1.01 ^a^	5.10 ± 1.12 ^a^
HP30	5.13 ± 1.00 ^ab^	4.96 ± 1.03 ^a^	4.90 ± 1.21 ^a^	4.83 ± 1.37 ^a^	4.83 ± 1.23 ^a^

Different superscript letters (^a–c^) show significant differences (*p* < 0.05) within the column.

## Data Availability

The original contributions presented in this study are included in the article. Further inquiries can be directed to the corresponding author.

## References

[1] Xu Y., Li J., Zhao J., Wang W., Griffin J., Li Y., Bean S., Tilley M., Wang D. (2021). Hempseed as a nutritious and healthy human food or animal feed source: A review. Int. J. Food Sci. Technol..

[2] Santos-Sánchez G., Álvarez-López A.I., Ponce-Espana E., Carrillo-Vico A., Bollati C., Bartolomei M., Lammi C., Cruz-Chamorro I. (2022). Hempseed (*Cannabis sativa*) protein hydrolysates: A valuable source of bioactive peptides with pleiotropic health-promoting effects. Trends Food Sci. Technol..

[3] Adesina I., Bhowmik A., Sharma H., Shahbazi A. (2020). A review on the current state of knowledge of growing conditions, agronomic soil health practices and utilities of hemp in the United States. Agriculture.

[4] Cherney J.H., Small E. (2016). Industrial hemp in North America: Production, politics and potential. Agronomy.

[5] Shen P., Gao Z., Fang B., Rao J., Chen B. (2021). Ferreting out the secrets of industrial hemp protein as emerging functional food ingredients. Trends Food Sci. Technol..

[6] Livadariu O., Raiciu D., Maximilian C., Căpitanu E. (2019). Studies regarding treatments of LED-s emitted light on sprouting hemp (*Cannabis sativa* L.). Rom. Biotechnol. Lett..

[7] Werz O., Seegers J., Schaible A.M., Weinigel C., Barz D., Koeberle A., Allegrone G., Pollastro F., Zampieri L., Grassi G. (2014). Cannflavins from hemp sprouts, a novel cannabinoid-free hemp food product, target microsomal prostaglandin E2 synthase-1 and 5-lipoxygenase. PharmaNutrition.

[8] Cabral E.M., Zhu X., Garcia-Vaquero M., Pérez-Vila S., Tang J., Gómez-Mascaraque L.G., Poojary M.M., Curtin J., Tiwari B.K. (2023). Recovery of Protein from Industrial Hemp Waste (*Cannabis sativa*, L.) Using High-Pressure Processing and Ultrasound Technologies. Foods.

[9] Montero L., Ballesteros-Vivas D., Gonzalez-Barrios A.F., Sánchez-Camargo A.d.P. (2023). Hemp seeds: Nutritional value, associated bioactivities and the potential food applications in the Colombian context. Front. Nutr..

[10] Banskota A.H., Tibbetts S.M., Jones A., Stefanova R., Behnke J. (2022). Biochemical characterization and in vitro digestibility of protein isolates from hemp (*Cannabis sativa* L.) by-products for salmonid feed applications. Molecules.

[11] Callaway J. (2004). Hempseed as a nutritional resource: An overview. Euphytica.

[12] House J.D., Neufeld J., Leson G. (2010). Evaluating the quality of protein from hemp seed (*Cannabis sativa* L.) products through the use of the protein digestibility-corrected amino acid score method. J. Agric. Food Chem..

[13] Karabulut G., Kahraman O., Pandalaneni K., Kapoor R., Feng H. (2023). A comprehensive review on hempseed protein: Production, functional and nutritional properties, novel modification methods, applications, and limitations. Int. J. Biol. Macromol..

[14] Alonso-Esteban J.I., Pinela J., Ćirić A., Calhelha R.C., Soković M., Ferreira I.C., Barros L., Torija-Isasa E., de Cortes Sánchez-Mata M. (2022). Chemical composition and biological activities of whole and dehulled hemp (*Cannabis sativa* L.) seeds. Food Chem..

[15] Zhang J., Griffin J., Li Y., Wang D., Wang W. (2022). Antioxidant properties of hemp proteins: From functional food to phytotherapy and beyond. Molecules.

[16] Sethi S., Tyagi S.K., Anurag R.K. (2016). Plant-based milk alternatives an emerging segment of functional beverages: A review. J. Food Sci. Technol..

[17] Parker H.W., Vadiveloo M.K. (2019). Diet quality of vegetarian diets compared with nonvegetarian diets: A systematic review. Nutr. Rev..

[18] Reid-McCann R.J., Brennan S.F., McKinley M.C., McEvoy C.T. (2022). The effect of animal versus plant protein on muscle mass, muscle strength, physical performance and sarcopenia in adults: Protocol for a systematic review. Syst. Rev..

[19] Friedman M., Brandon D.L. (2001). Nutritional and health benefits of soy proteins. J. Agric. Food Chem..

[20] Pinckaers P.J., Trommelen J., Snijders T., van Loon L.J. (2021). The anabolic response to plant-based protein ingestion. Sports Med..

[21] Talens C., Lago M., Simó-Boyle L., Odriozola-Serrano I., Ibargüen M. (2022). Desirability-based optimization of bakery products containing pea, hemp and insect flours using mixture design methodology. LWT.

[22] Nissen L., Demircan B., Taneyo-Saa D.L., Gianotti A. (2019). Shift of aromatic profile in probiotic hemp drink formulations: A metabolomic approach. Microorganisms.

[23] Dizlek H. (2015). Effects of amount of batter in baking cup on muffin quality. Int. J. Food Eng..

[24] Mildner-Szkudlarz S., Bajerska J., Górnaś P., Segliņa D., Pilarska A., Jesionowski T. (2016). Physical and bioactive properties of muffins enriched with raspberry and cranberry pomace powder: A promising application of fruit by-products rich in biocompounds. Plant Foods Hum. Nutr..

[25] Cho H.S., Olawuyi I.F., Park J.J., Lee W.Y. (2023). Quality characteristics of eggless muffins prepared using egg solution alternatives containing super mealworm protein isolate and carrageenan. Int. J. Food Sci. Technol..

[26] American Association of Cereal Chemists (2000). Approved Methods of the American Association of Cereal Chemists.

[27] Baek I.H., Cho H.S., Said N.S., Olawuyi I.F., Kim K.R., Lee W.Y. (2024). Physicochemical and nutritional characteristics of vegan protein bars formulated with sweet potato and rice protein. Int. J. Food Sci. Technol..

[28] Nielsen S.S. (2010). Phenol-sulfuric acid method for total carbohydrates. Food Analysis Laboratory Manual.

[29] Fitzgerald C., Gallagher E., Doran L., Auty M., Prieto J., Hayes M. (2014). Increasing the health benefits of bread: Assessment of the physical and sensory qualities of bread formulated using a renin inhibitory Palmaria palmata protein hydrolysate. LWT-Food Sci. Technol..

[30] Said N.S., Olawuyi I.F., Cho H.-S., Lee W.-Y. (2023). Novel edible films fabricated with HG-type pectin extracted from different types of hybrid citrus peels: Effects of pectin composition on film properties. Int. J. Biol. Macromol..

[31] Vl S. (1999). Analysis of total phenols and other oxidation substrates and antioxidants by means of Folin-Ciocalteu reagent. Methods Enzymol..

[32] Lowry O., Rosebrough N., Farr A.L., Randall R. (1951). Protein measurement with the Folin phenol reagent. J. Biol. Chem..

[33] Meilgaard M.C., Civille G.V., Carr B.T. (2007). Sensory Evaluation Techniques.

[34] Shanuke D.S., Edirisinghe EM RK B., Marapana RA U.J., Hettiarachi S. (2025). Development of omega-3 fortified stirred yoghurt with enhanced sensory and oxidative qualities through the addition of fish oil nanoemulsion and fruits. Int. J. Food Sci. Technol..

[35] (2012). Sensory Analysis—General Guidelines for the Selection, Training and Monitoring of Selected Assessors and Expert Sensory Assessors.

[36] El-Sohaimy S.A., Androsova N.V., Toshev A.D., El Enshasy H.A. (2022). Nutritional quality, chemical, and functional characteristics of hemp (*Cannabis sativa* ssp. sativa) protein Isolate. Plants.

[37] Zhou J., Liu J., Tang X. (2018). Effects of whey and soy protein addition on bread rheological property of wheat flour. J. Texture Stud..

[38] Liu M., Toth J.A., Childs M., Smart L.B., Abbaspourrad A. (2023). Composition and functional properties of hemp seed protein isolates from various hemp cultivars. J. Food Sci..

[39] Wendin K., Höglund E., Andersson M., Rothenberg E. (2017). Protein enriched foods and healthy ageing: Effects of protein fortification on muffin characteristics. Agro Food Ind. Hi-Tech.

[40] Höglund E., Albinsson B., Stuhr-Olsson G., Signäs M., Karlsson C., Rothenberg E., Wendin K. (2017). Protein and energy enriched muffins designed for nutritional needs of older adults. Nutr. Food Sci..

[41] Kwon Y., Kim K., Heo H., Lee J., Sung J. (2023). Vitamin E, phytosterol, and carotenoid contents of hemp (*Cannabis sativa* L.) seed. J. Korean Soc. Food Sci. Nutr..

[42] Aloo S.O., Park S., Oh D.-H. (2023). Impacts of germination and lactic acid bacteria fermentation on anti-nutrients, bioactive compounds, and selected functional properties of industrial hempseed (*Cannabis sativa* L.). Food Chem..

[43] de Souza E.C., Cordeiro D.A., Silva B.S., de Andrade Neves N., Schmiele M. (2022). Development of muffin with the incorporation of olive pomace flour, extra virgin olive oil and hydrolyzed soy protein. Res. Soc. Dev..

[44] Li N., Fan X., Chen T., Wang Y., Tan Z., Liu C., Zhou D., Li D. (2024). Molecular mechanism of color deepening of ready-to-eat shrimp during storage. Food Chem..

[45] Bosmali I., Kotsiou K., Matsakidou A., Irakli M., Madesis P., Biliaderis C.G. (2025). Fortification of wheat bread with an alternative source of bean proteins using raw and roasted Phaseolus coccineus flours: Impact on physicochemical, nutritional and quality attributes. Food Hydrocoll..

[46] Ye H., Zhang Y., Wang L., Ban J., Wei Y., Fan F., Guo B. (2024). Dynamic Study on Water State and Water Migration during Gluten–Starch Model Dough Development under Different Gluten Protein Contents. Foods.

[47] Ronda F., Oliete B., Gómez M., Caballero P.A., Pando V. (2011). Rheological study of layer cake batters made with soybean protein isolate and different starch sources. J. Food Eng..

[48] Zhao X., Xu S., Xu M., Li Y., Ji S., Wang F., Zhou Z., Wang Y., Shen J., Lu B. (2024). Mechanism of starch multi-scale structural in determining the textural properties and formability of starch pearls. Int. J. Biol. Macromol..

[49] Ma M., Guo H., Sun H., Sun J., Mu T. (2024). Improving the Quality Characteristics of Alum-Free Wet Starch Noodles: From the Perspective of Changing Types of Starches Used for Binder Pastes. ACS Food Sci. Technol..

[50] Huangfu J., Huang L., Gu Y., Yang S., Wu J., Liu T., Cai Y., Zhao M., Zhao Q. (2024). Effect of preheating-induced denaturation of proteins and oleosomes on the structure of protein and soymilk properties. Int. J. Biol. Macromol..

[51] Pathania S., Parmar P., Tiwari B.K. (2019). Stability of proteins during processing and storage. Proteins: Sustainable Source, Processing and Applications.

[52] Leistner L. (1992). Food preservation by combined methods. Food Res. Int..

[53] Kim S.-K. (1976). On bread staling with emphasis on the role of starch. Korean J. Food Sci. Technol..

[54] Chen C., Han Y., Li S., Wang R., Tao C. (2021). Nutritional, antioxidant, and quality characteristics of novel cookies enriched with mushroom (*Cordyceps militaris*) flour. CyTA-J. Food.

[55] Cheng Y.F., Bhat R. (2016). Functional, physicochemical and sensory properties of novel cookies produced by utilizing underutilized jering (*Pithecellobium jiringa* Jack.) legume flour. Food Biosci..

[56] Peleg M. (2019). The instrumental texture profile analysis revisited. J. Texture Stud..

[57] Shevkani K., Singh N. (2014). Influence of kidney bean, field pea and amaranth protein isolates on the characteristics of starch-based gluten-free muffins. Int. J. Food Sci. Technol..

[58] Ryu J.-H., Chung H.-J. (2018). Quality characteristics and antioxidant activity of rice cookies added with hempseed powder. Korean J. Food Nutr..

[59] Tang C.-H., Ma C.-Y. (2009). Heat-induced modifications in the functional and structural properties of vicilin-rich protein isolate from kidney (*Phaseolus vulgaris* L.) bean. Food Chem..

[60] Çabuk B. (2021). Influence of grasshopper (*Locusta migratoria*) and mealworm (*Tenebrio molitor*) powders on the quality characteristics of protein rich muffins: Nutritional, physicochemical, textural and sensory aspects. J. Food Meas. Charact..

[61] Bruttomesso M., Bianchi F., Pasqualoni I., Rizzi C., Simonato B. (2024). Evaluation of the technological and compositional features of pancakes fortified with Acheta domesticus. LWT.

[62] Pang J., Guan E., Yang Y., Li M., Bian K. (2021). Effects of wheat flour particle size on flour physicochemical properties and steamed bread quality. Food Sci. Nutr..

[63] Ozgolet M., Kasapoglu M.Z., Avcı E., Karasu S. (2024). Enhancing Gluten-Free Muffins with Milk Thistle Seed Proteins: Evaluation of Physicochemical, Rheological, Textural, and Sensory Characteristics. Foods.

[64] Kumar L.R., Sanath Kumar H., Tejpal C., Anas K., Nayak B., Sarika K., Greeshma S., Chatterjee N., Mathew S., Ravishankar C. (2021). Exploring the physical and quality attributes of muffins incorporated with microencapsulated squalene as a functional food additive. J. Food Sci. Technol..

[65] Mu T.-H., Zhang M., Sun H.-N., Pérez I.C. (2019). Sweet potato staple foods. Sweet Potato.

[66] Rouzbahani M., Hosseini H., Rastegar H., Zade S.V., Alizadeh A.M., Sharifan A., Hashempour-baltork F. (2024). Physicochemical, rheological and organoleptic characterizations of sponge cakes fortified with mycoproteins. J. Agric. Food Res..

[67] Dauthy M. (1995). Preservation by reduction of water content: Drying/dehydration and concentration (chapter 5.2). Fruit Veg. Process..

[68] Kaur G., Singh A., Khatkar S. (2024). Effect of ultrasonicated corn starch as a fat replacer on muffin quality and sensory characteristics. J. Food Meas. Charact..

[69] Xu J., Li Y., Zhao Y., Wang D., Wang W. (2021). Influence of antioxidant dietary fiber on dough properties and bread qualities: A review. J. Funct. Foods.

[70] Wang H., Liu W., Zhang P., Lian X. (2024). The Mechanism Underlying the Increase in Bread Hardness in Association with Alterations in Protein and Starch Characteristics During Room-Temperature Storage. Foods.

[71] Alhazmi H.A. (2019). FT-IR spectroscopy for the identification of binding sites and measurements of the binding interactions of important metal ions with bovine serum albumin. Sci. Pharm..

[72] Singh K.S., Majik M.S., Tilvi S. (2014). Vibrational spectroscopy for structural characterization of bioactive compounds. Comprehensive Analytical Chemistry.

[73] Aloo S.O., Park S., Jeong Y.-J., Gebre T.S., Oh D.-H. (2024). Characterization of different hempseed fractions after dehulling and defatting: Chemical composition and functional properties. J. Food Meas. Charact..

[74] Yoon H.-J., Park G.-H., Lee Y.-R., Lee J.-M., Ahn H.-L., Lee S.-O. (2023). Enzymatic preparation and antioxidant activities of protein hydrolysates from hemp (*Cannabis sativa* L.) seeds. Food Sci. Preserv..

[75] Khalesi M., Gcaza L., FitzGerald R.J. (2023). In Vitro Digestibility, Biological Activity, and Physicochemical Characterization of Proteins Extracted from Conventionally and Organically Cultivated Hempseed (*Cannabis sativa* L.). Molecules.

[76] Radünz M., Camargo T.M., Nunes C.F.P., Pereira E.D.S., Ribeiro J.A., Dos Santos Hackbart H.C., Radünz A.F.O., Radünz A.L., Gularte M.A., Da Fonseca Barbosa F. (2021). Gluten-free green banana flour muffins: Chemical, physical, antioxidant, digestibility and sensory analysis. J. Food Sci. Technol..

[77] Ouma F.O., Muriithi A.N., Anyango J.O. (2022). Nutritional composition and sensory Properties of wheat muffins enriched with Gonimbrasia zambesina, walker caterpillar flour. Int. J. Trop. Insect Sci..

[78] Ruiz G.A., Xiao W., van Boekel M., Minor M., Stieger M. (2016). Effect of extraction pH on heat-induced aggregation, gelation and microstructure of protein isolate from quinoa (*Chenopodium quinoa* Willd). Food Chem..

[79] Pihlanto A., Nurmi M., Pap N., Mäkinen J., Mäkinen S. (2021). The effect of processing of hempseed on protein recovery and emulsification properties. Int. J. Food Sci..

[80] Kamle M., Mahato D.K., Sharma B., Gupta A., Shah A.K., Mahmud M.C., Agrawal S., Singh J., Rasane P., Shukla A.C. (2024). Nutraceutical potential, phytochemistry of hemp seed (*Cannabis sativa* L.) and its application in food and feed: A review. Food Chem. Adv..

[81] Radočaj O., Dimić E., Tsao R. (2014). Effects of hemp (*Cannabis sativa* L.) seed oil press-cake and decaffeinated green tea leaves (*Camellia sinensis*) on functional characteristics of gluten-free crackers. J. Food Sci..

[82] Julakanti S., Charles A.P.R., Zhao J., Bullock F., Syed R., Myles Y., Wu Y. (2023). Hempseed protein (*Cannabis sativa* L.): Influence of extraction pH and ball milling on physicochemical and functional properties. Food Hydrocoll..

